# Serological Assessment for Celiac Disease in IgA Deficient Adults

**DOI:** 10.1371/journal.pone.0093180

**Published:** 2014-04-07

**Authors:** Ning Wang, Lennart Truedsson, Kerstin Elvin, Bengt A. Andersson, Johan Rönnelid, Lucia Mincheva-Nilsson, Annica Lindkvist, Jonas F. Ludvigsson, Lennart Hammarström, Charlotte Dahle

**Affiliations:** 1 Department of Laboratory Medicine, Karolinska Institutet at Karolinska University Hospital Huddinge, Stockholm, Sweden; 2 Department of Laboratory Medicine, Section of Microbiology, Immunology and Glycobiology, Lund University, Lund, Sweden; 3 Department of Medicine, Clinical Immunology and Allergy Unit, Karolinska Institutet, Stockholm, Sweden; 4 Department of Immunology, Sahlgrenska University Hospital, Gothenburg, Sweden; 5 Department of Immunology, Genetics and Pathology, Uppsala University, Uppsala, Sweden; 6 Department of Clinical Microbiology, Umeå University, Umeå, Sweden; 7 Department of Medical Epidemiology and Biostatistics, Karolinska Institutet, Stockholm, Sweden; 8 Department of Pediatrics, Örebro University Hospital, Örebro, Sweden; 9 Department of Clinical and Experimental Medicine, Faculty of Health Sciences, Linköping University, Linköping, Sweden; Instutite of Agrochemistry and Food Technology, Spain

## Abstract

**Purpose:**

Selective immunoglobulin A deficiency is the most common primary immunodeficiency disorder that is strongly overrepresented among patients with celiac disease (CD). IgG antibodies against tissue transglutaminase (tTG) and deamidated gliadin peptides (DGP) serve as serological markers for CD in IgA deficient individuals, although the diagnostic value remains uncertain. The aim of this study was to investigate the prevalence of these markers in a large cohort of IgA deficient adults with confirmed or suspected CD and relate the findings to gluten free diet.

**Methods:**

Sera from 488,156 individuals were screened for CD in seven Swedish clinical immunology laboratories between 1998 and 2012. In total, 356 out of 1,414 identified IgA deficient adults agreed to participate in this study and were resampled. Forty-seven IgA deficient blood donors served as controls. Analyses of IgG antibodies against tTG and DGP as well as HLA typing were performed and a questionnaire was used to investigate adherence to gluten free diet. Available biopsy results were collected.

**Results:**

Out of the 356 IgA deficient resampled adults, 67 (18.8%) were positive for IgG anti-tTG and 79 (22.2%) for IgG anti-DGP, 54 had biopsy confirmed CD. Among the 47 IgA deficient blood donors, 4 (9%) were positive for IgG anti-tTG and 8 (17%) for anti-DGP. Four were diagnosed with biopsy verified CD, however, 2 of the patients were negative for all markers. Sixty-eight of 69 individuals with positive IgG anti-tTG were HLA-DQ2/DQ8 positive whereas 7 (18.9%) of the 37 individuals positive for IgG anti-DGP alone were not.

**Conclusions:**

IgG anti-tTG seems to be a more reliable marker for CD in IgA deficient adults whereas the diagnostic specificity of anti-DGP appears to be lower. High levels of IgG antibodies against tTG and DGP were frequently found in IgA deficient adults despite adhering to gluten free diet.

## Introduction

Selective immunoglobulin A (IgA) deficiency is the most common primary immunodeficiency in Caucasians with a frequency of 1/600 in the general population. The current definition, established by the Pan-American Group for Immunodeficiency and the European Society for immunodeficiencies, defines the disorder as serum IgA levels below or equal to 0.07 g/L with normal serum levels of IgM and IgG in individuals of 4 years of age or older [1]. Two-thirds individuals with IgA deficiency are clinically asymptomatic, but the defect may be associated with recurrent respiratory and gastrointestinal tract infections and selected autoimmune disorders [2].

Celiac disease (CD) is a chronic immune-mediated disease which affects genetically susceptible individuals exposed to dietary gluten [3]. It is characterized by intraepithelial lymphocytosis, crypt hyperplasia and villous atrophy on a gluten-containing diet and mucosal recovery on a gluten free diet (GFD). The reported prevalence of CD varies widely, ranging from 0.3% to 2.4% in Europe [4]. However, the frequency of CD might be even higher as many individuals remain undiagnosed, probably due to the absence or atypical nature of symptoms [5].

There is a strong genetic predisposition in CD, where the major histocompatibility complex (MHC) region contributes about 40% of disease susceptibility [6], especially the human leukocyte antigen (HLA)-DQ2/DQ8 alleles, are present in more than 95% of the patients [7]. However, the presence of HLA-DQ2/DQ8 is a necessary, but not sufficient, prerequisite for development of CD [8].

In studies on small cohorts, IgA deficient patients have been shown to have a 10 to 20-fold increased risk of developing CD [9]–[11]. IgA deficiency has been reported to be strongly associated with the B8-DR3-DQ2 haplotype [12], [13], which is the strongest recognized risk haplotype for CD as well. CD is also associated with another IgA deficiency associated haplotype, DR7-DQ2, albeit to a much lesser degree [14], [15]. Taken together, this might explain the overlap between CD and IgA deficiency, indicative of a common genetic background.

The histological findings with small bowel mucosal villous atrophy and crypt hyperplasia have long been the gold standard for diagnosing CD. However, serological screening tests are valuable tools in selecting patients to undergo a diagnostic small bowel biopsy [16]. Initially, IgA anti-native gliadin antibodies (AGA) were widely used but have in recent years been replaced by anti-endomysial antibodies (EMA) which were found to be a higher specific diagnostic marker [17]. In 1997, tissue transglutaminase (tTG) was identified to be the auto-antigen targeted by EMA in patients with CD [18]. As the diagnostic accuracy of IgA antibodies against recombinant human tTG is high and markedly increased levels are highly predictive of CD [17], [19], [20], specific anti-tTG antibody tests have been widely used for CD screening. Recently, it has even been suggested that a small bowel biopsy is not necessary for the diagnosis of pediatric CD in symptomatic children with high levels of IgA anti-tTG [21].

Deamidated gliadin peptides (DGP) have been developed as the second generation anti-gliadin tests, including assessment of specific IgG antibodies [17]. This is a considerable improvement as tests based on detection of IgA antibodies alone yield false negative results in IgA deficient patients [22], [23]. Several reports have confirmed the increased diagnostic specificity of using DGP instead of native gliadin although the source of antigen and the performance may differ among the available commercial DGP assays [22], [24], [25]. However, Olen et al recently argued that anti-tTG is superior to anti-DGP for diagnosing CD since a combination of anti-tTG and anti-DGP did not provide a higher diagnostic accuracy than anti-tTG alone even in young children [26].

Several screening studies have been performed to evaluate CD serological markers in relation to small bowel biopsy findings among IgA deficient individuals with suspected and/or confirmed CD. However, the number of patients has been limited and mainly restricted to children (Table 1). In the present study, we recruited the hitherto, by far, largest cohort of adult IgA deficient individuals with suspected CD in order to estimate the prevalence of IgG antibodies against both tTG and DGP, and to investigate the correlation between different serological markers and the HLA types of the recruited individuals.

**Table 1:** Studies of IgG antibodies against endomysium (EMA), tissue transglutaminase (tTG), and deamidated gliadin peptides (DGP) among patients with celiac disease (CD) and IgA deficiency.

Ref	Year	Sample type	Sample Size	IgG anti-EMA(+)	IgG anti-tTG(+)	IgG anti-DGP(+)
[44]	1999	Children	2	2	—	—
[45]	2000	Adults and children	20	20	20	—
[46]	2002	Children	14	14	13	—
[31]	2003	Children	78	77	77	—
[47]	2003	NM^a^	11	4	0	—
[48]	2004	Children	11	—	11	—
[49]	2004	Adults and children	5	2	2 to 5	—
[50]	2005	Adults and children	115^b^	20	22	—
[51], [52]	2007	Adults and children	20	—	15 to 19^c^	—
[40]	2007	Children	2	—	2	2^d^
[53]	2008	Adults	2	—	2	—
[54]	2009	Adults and children	20	—	19	14
[39]	2009	Children	3	—	3	3^d^
[32]	2009	Children	3	—	3	3^d^
[55]	2010	Adults	24	19	23	21
		Children	10	6	10	10
[56]	2010	NM^a^	2	—	—	2
[57]	2010	Adults	4	—	3	3
[58]	2011	Adults and children	6	—	6	6
[59]	2012	NM^a^	4	—	4	4
[26]	2012	Children	5	—	NM	5
[35]	2012	Adults	22	—	18^e^	—
		Children	3	—	1	—
Current study		Adults	356	—	71	87

a NM: not mentioned.

b Patients with IgA deficiency and suspected CD.

c Based on different commercial tests.

d Positive for IgG anti-DGP antibodies (Euroimmun).

e Four patients had GFD at the time of sampling.

## Materials and Methods

### Ethics statement

The study was approved by Regionalaetikprövningsnämnden i Stockholm (Regional ethical review board in Stockholm) and Forkningsetikkommitte Syd, Karolinska Institutet (Research ethical committee South, Karolinska Institutet) which specifically encompassed the study presented in this manuscript (ethical permit numbers: 353/03; 356/03; 548/03; 04-261/4; 2008/1316-32; 2010/696-31/2; 2011/69-31/3). All adults have given their written informed consent to participate in the study.

### Study group

Originally blood samples from 488,156 individuals were referred to seven Swedish immunology centers between 1998 and 2012 for serological CD screening including measurements of total serum IgA levels. Altogether 1,414 adults were identified to be IgA deficient and 741 of them who could be traced were asked to submit new blood samples for follow-up testing. Of these, 356 individuals accepted to participate in this study. In addition, 47 IgA deficient blood donors from the blood transfusion center at the Karolinska University Hospital Huddinge, Sweden were included as controls. In total, 74 out of 356 patients and 6 out of 47 blood donors (n = 80) had undergone a small bowel biopsy and 58 (72.5%) were diagnosed with CD (54 patients and 4 blood donors).

### Celiac disease serological tests

Serum IgA levels of all IgA deficient individuals with suspected CD were determined by routine turbidimetric or nephelometric methods. Serum IgG anti-tTG and anti-DGP antibodies were analyzed in the newly collected serum samples at the clinical immunology unit at Linköping University Hospital, Sweden. Commercially available immunoassays for CD diagnostic testing were used according to the manufacturer instructions: (1) IgG antibodies against tTG (EliA™ Celikey®IgG, Phadia, Freiburg, Germany) were analyzed on ImmunoCAP 250 and the recommended cut-off <7 EliA U/mL was used. (2) Two different ELISAs were performed for detection of IgG antibodies against DGP including Gliadin IgG II (Quanta Lite™, INOVA Diagnostics, San Diego, CA, USA) and GAF-3X (Euroimmun, Lübeck, Germany). The recommended cut-offs 20 AU/mL (INOVA) and 25 RU/mL (Euroimmun) were used.

### HLA typing

HLA typing was performed at the clinical immunology laboratory, Karolinska University Hospital Huddinge, Sweden. DNA was extracted by standard procedures from whole blood samples of 355 IgA deficient individuals with suspected CD and 46 IgA deficient blood donors (2 samples were not available). All samples were subjected to whole genome amplification according to the manufacturer instructions (GenomePhi V2 DNA amplification kit, GE Healthcare, Buckinghamshire, UK) and subsequently HLA typed at the HLA-B, DR and DQ loci using polymerase chain reaction-single strand polymorphism (PCR-SSP) [27]. The kits used included HLA-B low resolution, DQ-DR SSP Combi Trays and DQB1*03 high resolution kits (Olerup SSP AB, Stockholm, Sweden).

### Questionnaire

A questionnaire regarding GFD was mailed to 359 IgA deficient individuals (44 individuals from one clinic were excluded due to ethical constraints).

### Statistics

As our subgroups were of unequal sizes and the levels of different serological markers in different subgroups showed unequal variances, we used Welch's *t* test, which is an adaptation of Student's *t* test. For comparison of numbers of individuals in different groups, Chi-squared test was used. Statistical significance was accepted as *p*<0.05.

## Results

### Serological marker levels

**Figure 1:**
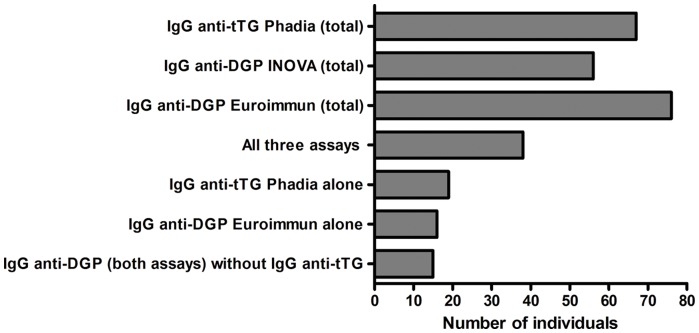
Number of IgA-deficient individuals testing positive in each assay.

IgA deficiency (serum IgA≤0.07 g/L) was confirmed in all resampled individuals with suspected CD and blood donors. Out of the IgA deficient individuals with suspected or confirmed CD (n = 302 and 54 respectively), 67 (18.8%) were positive for IgG anti-tTG antibodies, 56 (15.7%) for IgG anti-DGP with the INOVA assay and 76 (21.3%) with the Euroimmun assay. In total, 38 of these individuals (10.7%) were positive for all three serological markers and 108 individuals were positive for at least one marker (Figure 1). In the IgA deficient blood donor group (n = 47), 4 (8.5%) were positive for IgG anti-tTG, 4 (8.5%) and 8 (17.0%) were positive for IgG anti-DGP using the INOVA assay and the Euroimmun assay respectively, whereas only 2 individuals (4.8%) were positive in all three assays (Table 2). In total, significantly more samples were positive for IgG anti-DGP with the Euroimmun assay (n = 84) than with the INOVA assay (n = 60) (*p*<0.001, Chi-squared *t* test). Interestingly, there was no individual with positive IgG anti-DGP (INOVA) alone (Figure 1). In our cohort, 39 of the 71 IgG anti-tTG positive individuals had very high antibody levels (>10 times above cut-off) even including 16 patients reporting to be on a GFD. Out of the 58 individuals (54 patients and 4 blood donors) with biopsy confirmed CD, 34 (63%) were positive for IgG anti-tTG, 28 (52%) for IgG anti-tTG with the INOVA and 35 (65%) with the Euroimmun assay at the time of resampling.

**Table 2:** Numbers of individuals with elevated levels of IgG antibodies against tissue transglutaminase (tTG) and/or deamidated gliadin peptides (DGP), HLA-DQ2/DQ8, celiac disease (CD) and gluten free diet (GFD) among patients and blood donors with IgA deficiency (subgroups without positive serology are not shown).

Group	IgG anti-tTG (Phadia)	IgG anti-DGP (INOVA)	IgG anti-DGP (Euroimmun)	Total No.	Biopsy confirmed CD	DQ2/DQ8	Non-DQ2/DQ8
GFD (n = 36/67)	**+**	**−**	**−**	8	5	8	0
	**−**	**−**	**+**	3	2	3	0
	**+**	**+**	**−**	1	0	1	0
	**+**	**−**	**+**	2	2	2	0
	**−**	**+**	**+**	6	5	6	0
	**+**	**+**	**+**	16	7	16	0
Non GFD (n = 38/184)	**+**	**−**	**−**	5	3	5	0
	**−**	**−**	**+**	7	0	5	2
	**+**	**+**	**−**	1	0	1	0
	**+**	**−**	**+**	4	1	4	0
	**−**	**+**	**+**	6	0	5	1
	**+**	**+**	**+**	15	7	14*	0
No reply (n = 10/61)	**+**	**−**	**−**	4	1	4	0
	**−**	**−**	**+**	5	0	2	3
	**+**	**+**	**+**	1	0	1	0
Not available (n = 13/38)	**+**	**−**	**−**	2	1	2	0
	**−**	**−**	**+**	1	1	1	0
	**+**	**+**	**−**	1	0	1	0
	**+**	**−**	**+**	1	1	1	0
	**−**	**+**	**+**	3	2	3	0
	**+**	**+**	**+**	5	4	5	0
Blood donors (n = 10/47)	**+**	**−**	**−**	2	0	0*	1
	**−**	**−**	**+**	4	0	3	1
	**−**	**+**	**+**	2	1	2	0
	**+**	**+**	**+**	2	1	2	0

* One DNA sample in this group was not available (total number: 2).

+ represents positive result.

− represents negative result.

### Genetic correlation

**Figure 2:**
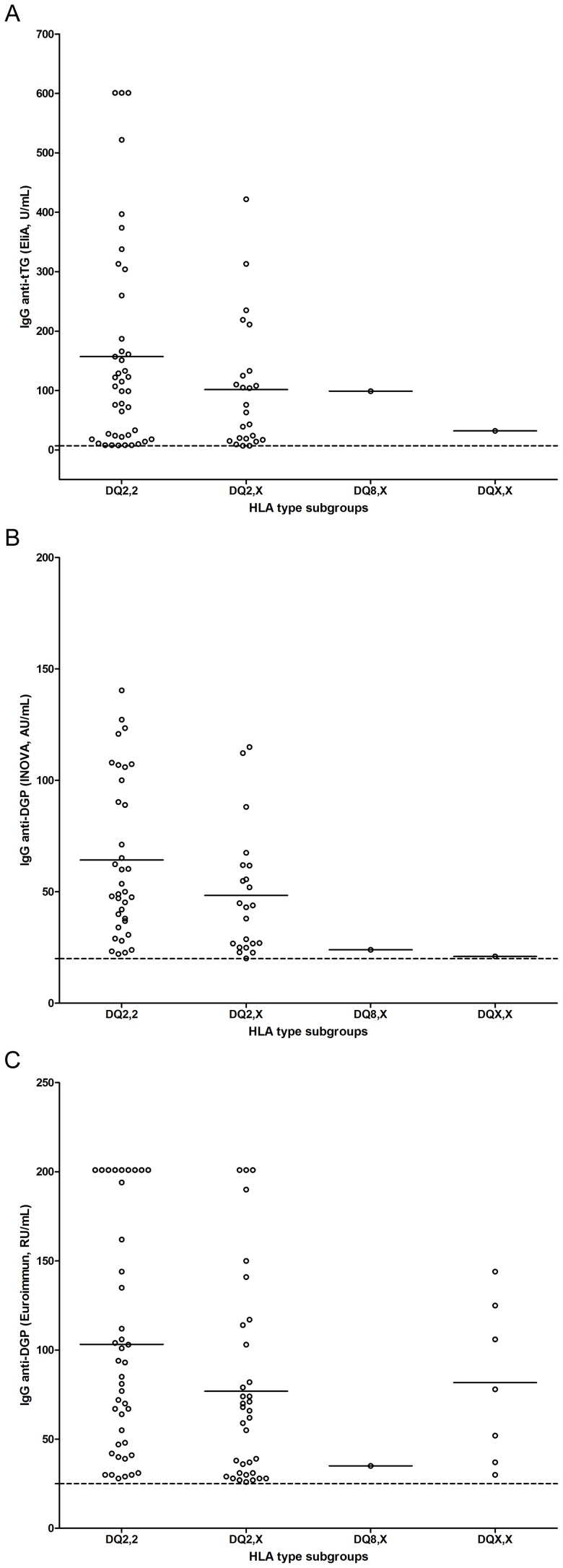
HLA-DQ2/DQ8 among individuals with IgA deficiency and elevated levels of IgG antibodies against tissue transglutaminase (tTG) (2a) and deamidated gliadin peptides (DGP) (2b and 2c). DQ2,2 represents individuals homozygous for DQ2 (n = 42, 35 and 41 respectively in each subfigure); DQ2,X represents individuals heterozygous for DQ2 (n = 24, 22 and 34 respectively in each subfigure); DQ8,X represents individuals heterozygous for DQ8 (including one individual homozygous for DQ8) (one positive sample in each subfigure); DQX,X represents individuals carried non DQ2/DQ8 alleles (n = 1, 1 and 7 respectively in each subfigure). The dotted lines represent the recommended cut-off for seropositivity and the short bars represent the mean values for each group.

Our cohort was divided into different subgroups based on HLA types, including those homozygous for DQ2 (DQ2,2), heterozygous for DQ2 (DQ2,X), those carrying DQ8 but not DQ2 (DQ8,X which includes one individual homozygous for DQ8) and non-DQ2/DQ8 alleles (DQX,X). The elevated antibody levels in different HLA subgroups were compared for each assay (Figure 2a, Figure 2b and Figure 2c). Individuals homozygous and heterozygous for DQ2 represented the majority of samples with elevated antibody levels in all three assays. There was no significant difference of the antibody levels in homozygous/heterozygous DQ2 subgroups regarding IgG anti-tTG and anti-DGP with the INOVA assay (*p* = 0.151 and 0.054 respectively). The levels of IgG anti-DGP with the Euroimmun assay were significantly higher in the homozygous DQ2 subgroup as compared to the heterozygous DQ2 subgroup (*p* = 0.032, Welch's *t* test). Individuals carrying DQ8 or non-DQ2/DQ8 alleles showed markedly lower antibody levels than those carrying DQ2 with exception for the Euroimmun assay where there was no significant difference between the antibody levels in the non-DQ2/DQ8 (DQX,X) compared to the DQ2 subgroups (*p* = 0.114 and 0.807 respectively, Welch's *t* test, Figure 2c).

DNA samples from 69/71 individuals (66 patients and 3 blood donors) with positive IgG anti-tTG (with or without positive anti-DGP) were available for HLA typing and 68 (66 patients and 2 blood donors) were DQ2/DQ8 positive (Table 2). Out of the 37 individuals that were only positive for anti-DGP, 5 carried neither DQ2 nor DQ8 and 4 of the latter were only positive for anti-DGP using the Euroimmun assay.

The association between positive antibody assays and the common IgA deficiency risk haplotypes (DR3-DQ2, DR7-DQ2 and DR1-DQ5) was also analyzed regarding to having GFD or not (Table 3). Most of the enrolled individuals carried at least one of the three major IgA deficiency risk haplotypes, mostly the DR3-DQ2 haplotype.

**Table 3:** Numbers of individuals with elevated levels of IgG antibodies against tissue transglutaminase (tTG) and/or deamidated gliadin peptides (DGP), and IgA deficiency associated HLA haplotypes among IgA deficient patients with suspected celiac disease (CD) with or without GFD.

Group	IgG anti-tTG (Phadia)	IgG anti-DGP (INOVA)	IgG anti-DGP (Euroimmun)	Total No.	DR3-DQ2 haplotype	DR7-DQ2 haplotype	DR1-DQ5 haplotype	Other haplotype
GFD (n = 36)	**+**	**−**	**−**	8	13	1	1	1
	**−**	**−**	**+**	3	3	1	0	2
	**+**	**+**	**−**	1	1	0	0	1
	**+**	**−**	**+**	2	2	0	1	1
	**−**	**+**	**+**	6	8	1	0	3
	**+**	**+**	**+**	16	20	3	4	5
Non GFD (n = 34)	**+**	**−**	**−**	5	6	2	0	2
	**−**	**−**	**+**	7	6	1	3	4
	**+**	**+**	**−**	1	0	0	0	2
	**+**	**−**	**+**	4	7	0	1	0
	**−**	**+**	**+**	6	5	2	3	2
	**+**	**+**	**+**	15	21	4	0	3

### Small bowel biopsy test and questionnaire results

**Figure 3:**
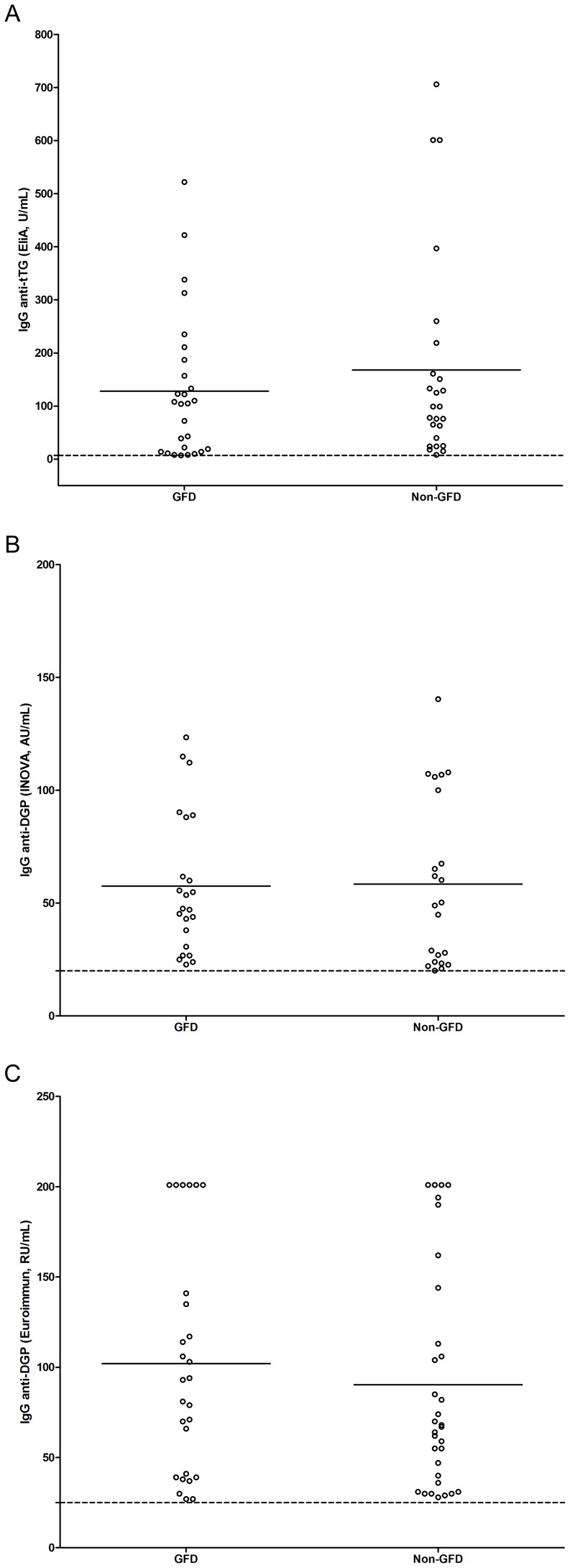
Elevated levels of IgG antibodies against tissue transglutaminase (tTG) (3a) and deamidated gliadin peptides (DGP) (3b and 3c) in relation to gluten free diet (GFD) among IgA deficient individuals with suspected celiac disease (CD). GFD represents individuals reporting to adhere to GFD (n = 27, 23 and 27 respectively in each subfigure); non-GFD represents individuals not adhering to GFD (n = 25, 22 and 32 respectively in each subfigure). The dotted lines represent the recommended cut-off for seropositivity and the short bars represent the mean values for each group.

Since our 58 biopsy confirmed CD patients (54 from the patient group and 4 from the blood donor group) showed varying antibody levels, questionnaires were sent to the individuals for information on dietary restriction (GFD). Completed questionnaires were received from 261 out of 359 patients (6 individuals had died after re-sampling) showing that 68 reported to be on a GFD whereas 193 reported not. Figure 3 shows the distribution of IgG antibody levels among patients with/without GFD. Surprisingly, there was no significant difference between the two groups (*p* = 0.408, 0.938 and 0.476 respectively in three assays, Welch's *t* test).

**Figure 4:**
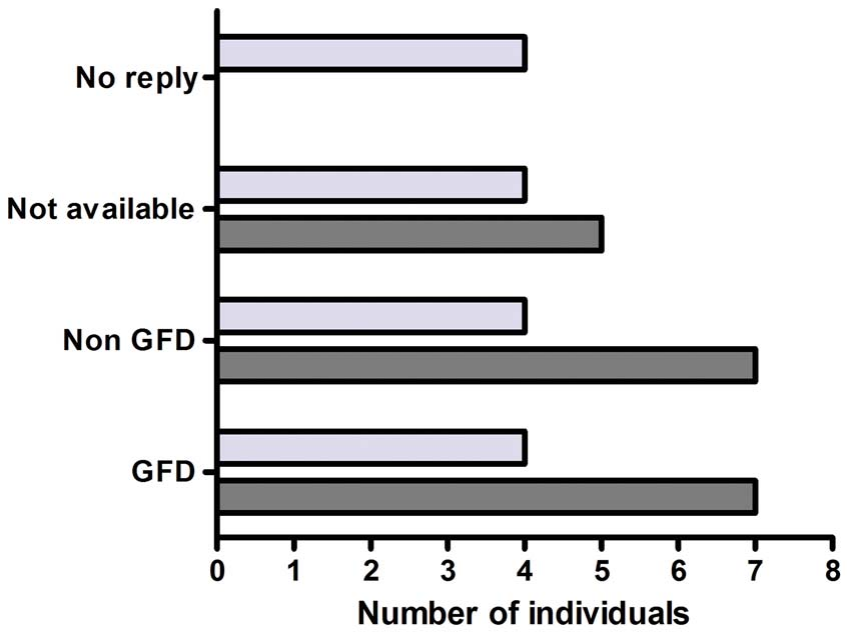
Numbers of IgA deficient individuals with biopsy confirmed celiac disease positive (dark grey bars) or negative (light grey bars) for IgG antibodies against tissue transglutaminase (tTG) and deamidated gliadin peptides (DGP) in relation to gluten free diet (GFD).

Twenty of the biopsy confirmed CD patients were positive in all IgG antibody assays, seven of whom reported to adhere to a GFD and seven not (one patient had died and 5 were not sent questionnaires due to ethical constraints). There were 16 confirmed CD patients negative for all markers and only four of them reported being on a GFD (4 were still on a gluten containing diet, 4 did not reply to the questionnaire and 4 were not sent questionnaires due to ethical constraints) as shown in Figure 4. Fifty-four out of 58 biopsy verified CD patients carried HLA-DQ2/DQ8 (one DNA sample was not available). Interestingly, 45 of the 54 confirmed CD cases from the patient group carried at least one copy of the B8-DR3-DQ2 haplotype, which is a common IgA deficiency risk haplotype and contains the CD risk allele DQ2. Moreover, 16 of 45 patients carried two copies of the B8-DR3-DQ2 haplotype. Two of the 4 confirmed CD patients from the blood donor group carried one copy of the B8-DR3-DQ2 haplotype and one individual carried the other CD risk allele (DQ8).

## Discussion

IgA deficiency is the most common primary immunodeficiency in Caucasians. IgA deficient individuals demonstrate a higher incidence of silent forms of CD [28] and a 10 to 20-fold increased risk of developing overt CD [9]–[11], thus highlighting the need to evaluate CD diagnostic antibodies of the IgG class rather than the IgA class. However, to date only a limited number of cases have been investigated for CD related IgG antibodies, mainly restricted to children (Table 1). Therefore, we investigated the prevalence of IgG antibodies against both tTG and DGP, the latter including two commercial kits (INOVA and Euroimmun), in a large cohort of IgA deficient adults, detected by routine serological screening for CD.

In IgA competent individuals, high IgA anti-tTG levels (>10 times above cut-off) are strongly associated with CD as reported by Vermeersch *et al* as they found likelihood ratios of >100 for CD among individuals with high levels of IgA anti-tTG [29]. In children, a small bowel biopsy is no longer considered to be required for diagnosing CD provided that the individuals also carry CD risk HLA alleles and have symptoms suggestive of CD [30]. However, whether this is true regarding high levels of IgG anti-tTG in individuals with IgA deficiency is still unknown. One striking finding in our cohort was that more than half of the IgG anti-tTG positive individuals had very high levels (>10 times above cut-off), even including 16 patients reporting to be on a GFD.

Already in 2003, Korponay-Szabo and coworkers [31] noted that the decrease in IgG anti-tTG antibody levels was slow in CD patients with IgA deficiency and most of them were still positive after more than two or three years on a GFD, in contrast to IgA competent CD patients where levels decreased markedly or returned to normal for IgG anti-tTG/DGP antibodies after one year on a GFD [32]–[34]. Recently, Chow et al [35] also reported that only half of their CD patients with IgA deficiency had normalized their serology after having a GFD for a mean of 7.25 years.

We observed that there was no difference in IgG antibody levels in patients with/without GFD (Figure 3). High levels of IgG anti-tTG and/or anti-DGP in patients who did not adhere to a GFD (n = 184) may reflect undiagnosed CD patients. However, 36 of 67 enrolled IgA deficient patients with suspected CD on a GFD were still positive for at least one marker, 27 of whom were positive for IgG anti-tTG antibodies and 16 tested positive in all three assays. These data are supported by a previous study showing persistent small bowel villous atrophy, defined as Marsh class 3, in 43% of CD patients when they were re-biopsied 0,5–5 years after diagnosis [36]. The explanations for this could be that, despite being diagnosed with CD, these patients were not motivated for a strict GFD, presumably due to a combination of mild gastrointestinal symptoms and that adhering to GFD is expensive and difficult in a social context; second, consistent with previous publications, levels of IgG antibodies appear to decrease slowly in IgA deficient patients with CD despite being on a GFD for a long period.

In our 27 patients who were positive for IgG anti-tTG antibodies and adhering to a GFD, 26 carried at least one copy of the B8-DR3-DQ2 haplotype and one did not carry the complete haplotype but still carried DR3-DQ2 (Table 3). This might be part of the immune-regulatory defect seen in IgA deficient individuals, as individuals carrying the B8-DR3-DQ2 haplotype have alterations in their immune responses, regardless of their IgA levels [37]. Thus, it is still not clear if the slower decrease in IgG anti-tTG antibodies is directly due to IgA deficiency itself or its associated HLA haplotype.

HLA types were obtained in 401 IgA deficient individuals (two samples were not available). Individuals carrying DQ2 were markedly overrepresented among the individuals who were positive for IgG antibodies to CD related antigens and also showed considerably higher IgG antibody levels as compared to other subgroups (Figure 2), which is in concordance with another study on IgA competent adult CD patients positive for IgA anti-EMA [38], suggesting that the genetic association between DQ2 and CD might be the same in IgA deficient and IgA sufficient adults. With exception for the Euroimmun assay, individuals carrying DQ8 not DQ2 showed very low IgG antibody levels, supporting the notion that the DQ2 allele itself may play a main role for the induction of these antibody specificities.

In 2009, Prause et al [39] claimed that the high accuracy of IgG anti-DGP (Euroimmun) might offer a chance to detect CD in IgA deficient patients without further diagnostic efforts as three IgA deficient children with negative IgA anti-EMA and anti-tTG, had high concentrations of IgG anti-tTG and anti-DGP (Euroimmun). Other studies have also shown that IgG anti-DGP antibodies using the Euroimmun assay were of value for diagnosing CD in IgA deficient children though the authors suggested that further studies were necessary to confirm the diagnostic accuracy of the assay in IgA deficiency [32], [40]. As shown in Table 2, significantly more samples were positive for IgG anti-DGP with the Euroimmun assay than with the INOVA assay and, six out of 20 individuals who tested only positive for IgG anti-DGP with the Euroimmun assay were DQ2/DQ8 negative (1 out of 7 DQ2/DQ8 negative patients was positive in both IgG anti-DGP assays). This implies that the diagnostic accuracy of IgG anti-DGP in the Euroimmun assay is questionable in IgA deficient adults.

A 3-fold higher frequency of IgA deficiency was found in our cohort (approximately 1 in 200, including 1180 IgA deficient children) as compared to expected in the general population (1/600) and it is likely to reflect the higher prevalence of gastrointestinal problems, including CD, in IgA deficient individuals. In addition, a recent study showing that individuals with IgA deficiency were at higher relative mortality risks in the first 10–15 years after diagnosis [41], potentially associated with the higher mortality noted in patients with CD, underlines the need for a reliable diagnostic markers of the IgG class. To date, IgG anti-tTG antibody seems to be the most reliable marker and the importance of isolated presence of IgG anti-DGP is still unknown in IgA deficient cases. In our study, almost all cases with IgG anti-tTG positive levels also carried the CD risk alleles, that was not the case for individuals with an isolated presence of IgG anti-DGP, again indicating a rather lower diagnostic specificity.

Approximately 500,000 individuals were initially screened for CD related antibodies and serum IgA levels in Sweden during 1998–2012, indicating that more than 5% of the Swedish population was suspected of having CD during this period, which is markedly higher than the published prevalence of CD in other European populations [42]. Furthermore, this shows that serological CD screening is liberally performed and that awareness is high among physicians that IgA anti-tTG is a reliable marker for CD. Our results suggest that many individuals with IgA deficiency, who may have CD or latent CD could have escaped further investigations with IgG antibody screening and/or small bowel biopsy test and may therefore not be properly diagnosed. Thus, it seems not to be equally recognized well among clinicians, not specialized in gastroenterology, that the IgG anti-tTG antibody is a strong diagnostic CD marker in IgA deficient individuals, conferring a major risk to underdiagnosed CD in such cases. This assumption is supported by the fact that we have small bowel biopsy results in only 80 of 403 enrolled IgA deficient individuals.

It is the laboratory's responsibility to screen for IgA deficiency in all samples referred for CD screening and to inform the referring clinicians that a negative result for CD markers of the IgA class is only valid provided that the individual does not have IgA deficiency. Löwbeer and Wallinder [43] recently reported that testing total IgA levels in the screening of CD was unnecessary by using the EliA Celikey assay since their results showed that samples with IgA deficiency presented very low values in the IgA anti-tTG test. This is also consistent with our own experience when analyzing serum samples from 238 IgA deficiency individuals for IgA anti-tTG antibodies using the EliA Celikey assay since all tested sera gave very low responses (unpublished data). This procedure may facilitate the CD screening laboratory routine by reducing the number of samples needed to be analyzed for total serum IgA levels.

Due to the ethical constraints, we were not be able to follow up all recruited individuals and information about small bowel biopsy results were only available in few IgA deficient subjects, limiting the solidity of our conclusion. However, our findings suggest that, although within the limitations of a retrospective questionnaire on dietary restrictions (261/359), the diagnostic values differ between different commercial IgG anti-DGP assays and many CD patients continue to eat gluten containing food. Furthermore, 4 of 6 our IgA deficient blood donors who had undergone small bowel biopsy were found to have CD, underlining the increased prevalence of silent CD in IgA deficient individuals. Thus, awareness of CD among IgA deficient individuals still needs to be increased in order to promote strict GFD, even in clinically silent cases, thereby preventing long term serious clinical consequences.
